# Supplementary material for: Thumb-domain dynamics modulate the functional repertoire of DNA-Polymerase IV (DinB)
Source: Nucleic Acids Res. 2023 Jun 1;51(13):7036–52. doi: 10.1093/nar/gkad490 (PMC10359629; doi:10.1093/nar/gkad490)
Supplement: gkad490_Supplemental_File [file gkad490_supplemental_file.pdf]

SUPPLEMENTARY INFORMATION FOR

Thumb-domain dynamics modulate the functional repertoire of DNA-Polymerase IV (DinB)

Damasus C. Okeke[1,2], Jens Lidman[1,2], Irena Matečko-Burmann[1,3], Björn M. Burmann[1,2]

[1]Department of Chemistry and Molecular Biology, University of Gothenburg, 405 30 Göteborg, Sweden

[2]Wallenberg Centre for Molecular and Translational Medicine, University of Gothenburg, 405 30 Göteborg, Sweden

[3]Department of Psychiatry and Neurochemistry, University of Gothenburg, 405 30 Göteborg, Sweden

*Correspondence should be addressed to BMB:

Tel: +46-317863937; Email: bjorn.marcus.burmann@gu.se

Supplementary Table S1: Primers used in this study to generate the different DinB constructs used.

Construct	Primers (5´– 3´)	Plasmid
**DinB**	(purchased from GenScript)	pET28_DinB
**DinB-NTD**	Forward: GGCCAGTTTGTGATTACGTAGGCAGAAGTTCCGGCATT<br>Reverse: AATGCCGGAACTTCTGCCTACGTAATCACAAACTGGCC	pET28b_DinB-NTD
**DinB-Palm**	Forward_1: GCATCTGGAACAGATTGGCGGTATGCGTAAAATCATTCATGTG<br>Reverse_1: GTGGTGGTGGTGGTGCTCGAGGTCCATATCCACATGAATGATTTTAC<br>Forward_2: CATTCATGTGGATATGGACGGGCGCTTTGACGCCTACAAAG<br>Reverse_2: GTGGTGGTGGTGGTGCTCGAGTCACGTAATCACAAACTGGCCGTTG	pET28b_DinB-Palm
**DinB-Fingers**	Forward: GCATCTGGAACAGATTGGCGGTTGCTTTTCGCCGCAGTGGAG<br>Reverse: GTGGTGGTGGTGGTGGTGCTCGAGTTACGGAAGCAAGGTGAGATG	pET28b_DinB-Fingers
**DinB-Thumb**	Forward: GCATCTGGAACAGATTGGCGGTACGCCGGCAGAAGTTCCGGC<br>Reverse: GGTGGTGGTGGTGGTGCTCGAGTCATTTTCGCAACCGTTCGCTG	pET28b-Thumb
**DinB-PAD**	Forward: CATCTGGAACAGATTGGCGGTGTTAACATCGCGTTCGTCAATCCC<br>Reverse: ACCGCCAATCTGTTCCAGATGGGGATTGACGAACGCGATGTTAAC	pET28b-DinB-PAD
**DinBΔPAD**	Forward: GTAGTCAGGGGATTGACTAACGCGATGTTAACAGC<br>Reverse: GCTGTTAACATCGCGTTAGTCAATCCCCTGACTAC	pET28bΔDinB-PAD

Supplementay Table S2: Conditions used for the NMR experiments for the different DinB constructs used in this study.

Protein	NMR buffer
**DinB**	50 mM KPi, 300 mM KCl, 50 mM Arginine, 50 mM Glutamine, 1 mM EDTA, 1 mM TCEP, 1 mM CHAPSO, pH 6.5
**DinBΔPAD**	PBS, 50 mM Arginine, 50 mM Glutamine, 1 mM EDTA, 1 mM TCEP, 0.1 mM CHAPSO, pH 7.4
**DinB-PAD**	50 mM KPi, 100 mM KCl, 50 mM Arginine, 50 mM Glutamine, 1 mM EDTA, 1 mM TCEP, 0.1 mM CHAPSO, pH 6.8
**DinB-Thumb**	50 mM KPi, 300 mM KCl, 50 mM Arginine, 50 mM Glutamine, 1 mM EDTA, 1 mM TCEP, 0.1 mM CHAPSO, pH 7.5

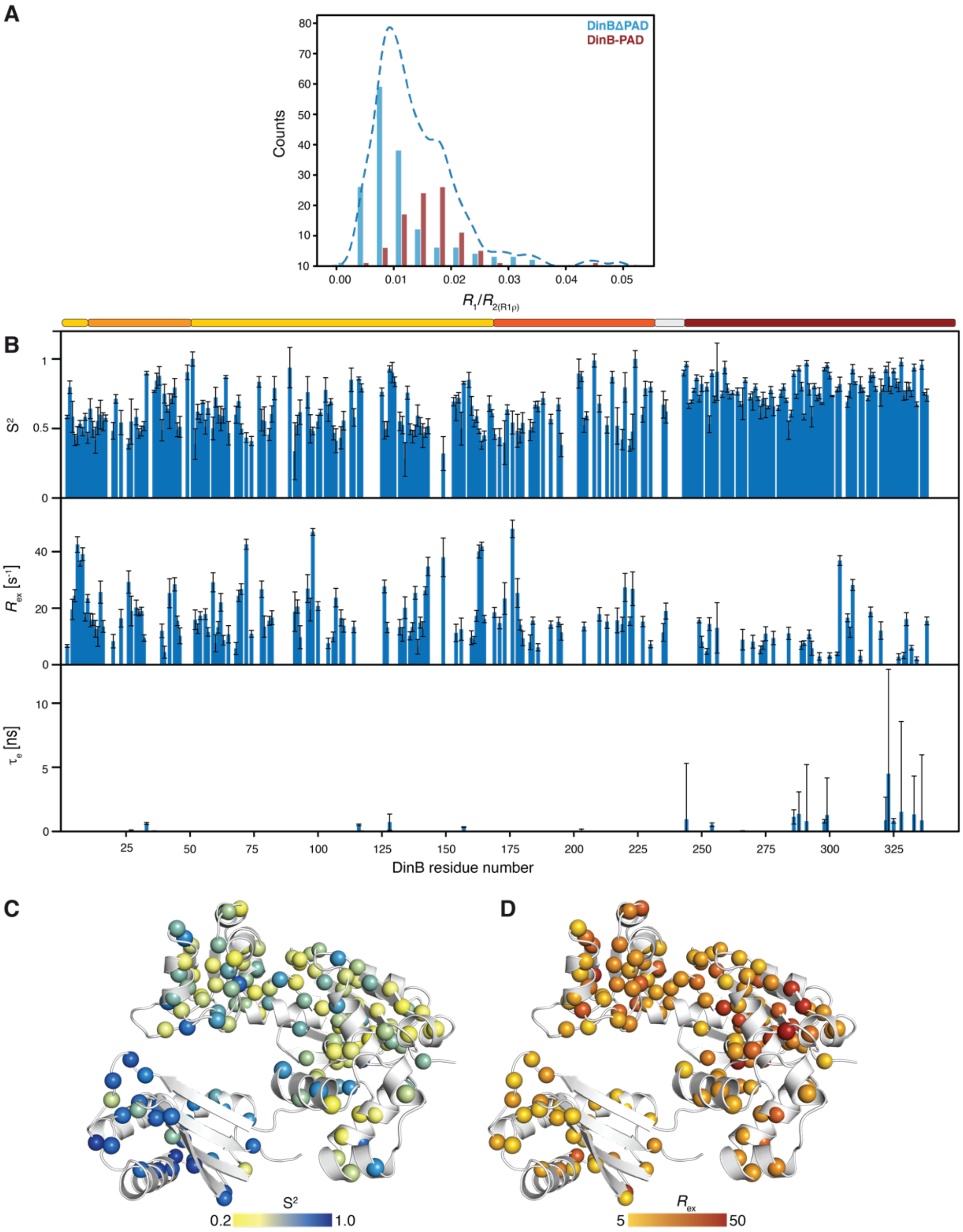

Supplementary Figure S1. A) Histogram of $R_1/R_{2(R1\rho)}$ values of the DinBΔPAD (blue) and DinB-PAD (red). The dashed line corresponds to a kernel density estimation (KDE) of both domain values using a bandwidth of h=0.5. The bimodal distribution indicates partially decoupled motion of the DinB-PAD within full-length DinB. **B**) The generalized order parameter S^2 (top), the conformational exchange contributions, R_{ex}, and the τ_e correlation time reporting on fast internal motions are plotted against the DinB residue number. **C**) S^2 values obtained for DinB plotted on the DinB structure (PDB-ID: 4Q45). The amide moieties are shown as spheres and the S^2 values are indicated by the yellow to blue gradient. **D**) Residues exhibiting conformational exchange on the micro- to millisecond timescale are plotted on

the DinB structure (PDB-ID: 4Q45). The amide moieties are shown as spheres and the R_{ex} values are indicated by the yellow to red gradient.

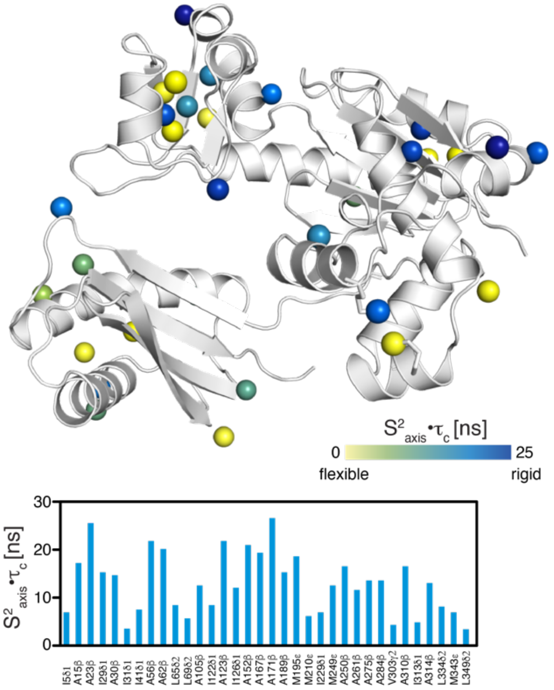

Supplementary Figure S2. Local methyl group dynamics on the pico– to nanosecond timescale probed by methyl single-quantum (SQ) and triple-quantum (TQ) relaxation experiments showing the product of the local order parameter and the overall tumbling constant, S^2_{axis}•τ_C. the methyl groups are shown as spheres and the obtained S^2_{axis}•τ_C-values by a yellow to blue gradient (top). S^2_{axis}•τ_C-values plotted against the DinB amino acid sequence (bottom).

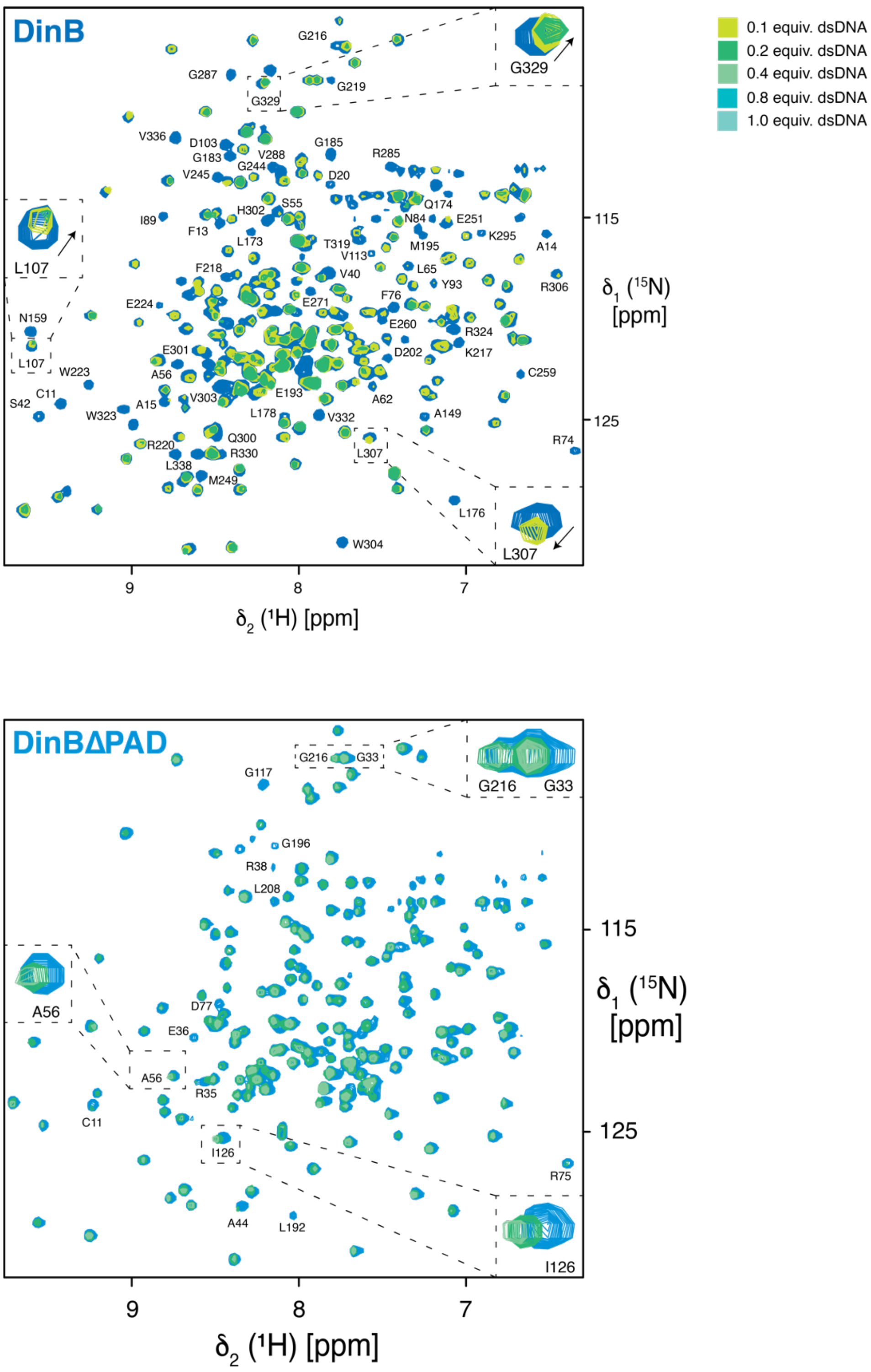

Supplementary Figure S3. Titration of increasing amounts of dsDNA to either [U-^{2}H,^{15}N]-DinB and [U-^{2}H,^{15}N]-DinBΔPAD. Overlay of 2D [^{15}N,^{1}H]-NMR spectra of the two different DinB constructs in the absence (blue) and in the presence of increasing amounts of DNA as indicated by the green gradient acquired in NMR-buffer at 298 K. Related to **Figure 3A**.

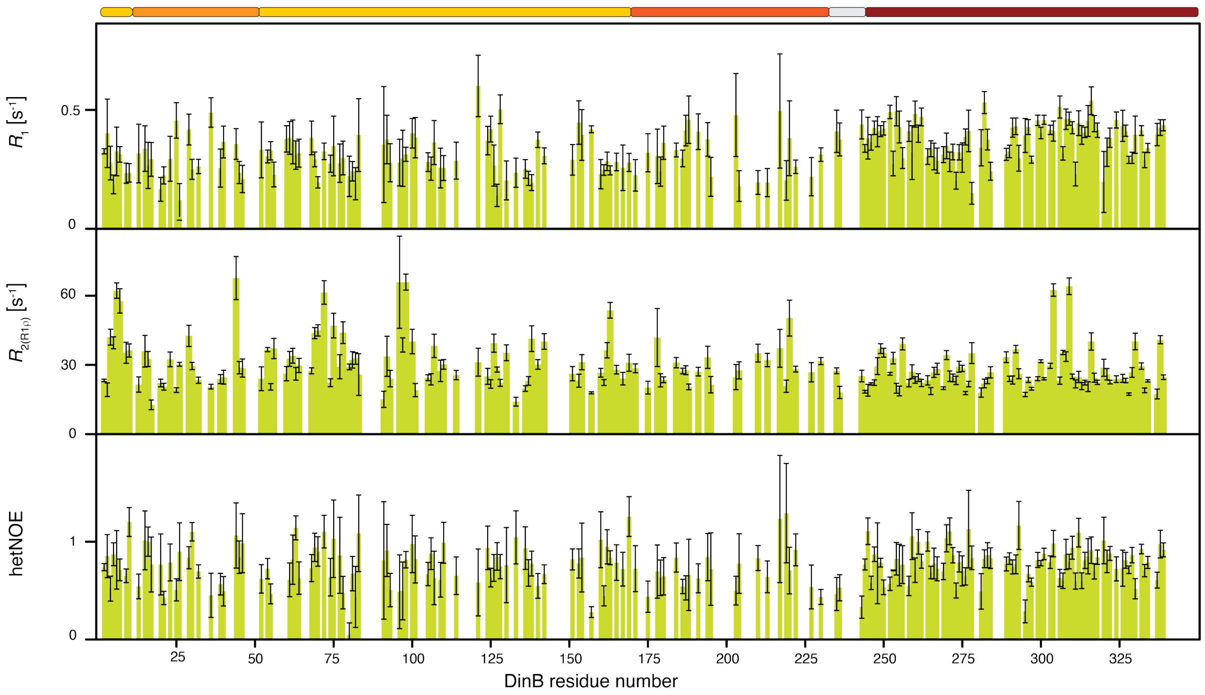

Supplementary Figure S4. Backbone relaxation of DinB in the presence of dsDNA. The hetNOE (top), the longitudinal relaxation rate R_1 (middle), and the $R_{2(R1\rho)}$ (bottom) are plotted against the DinB residue number.

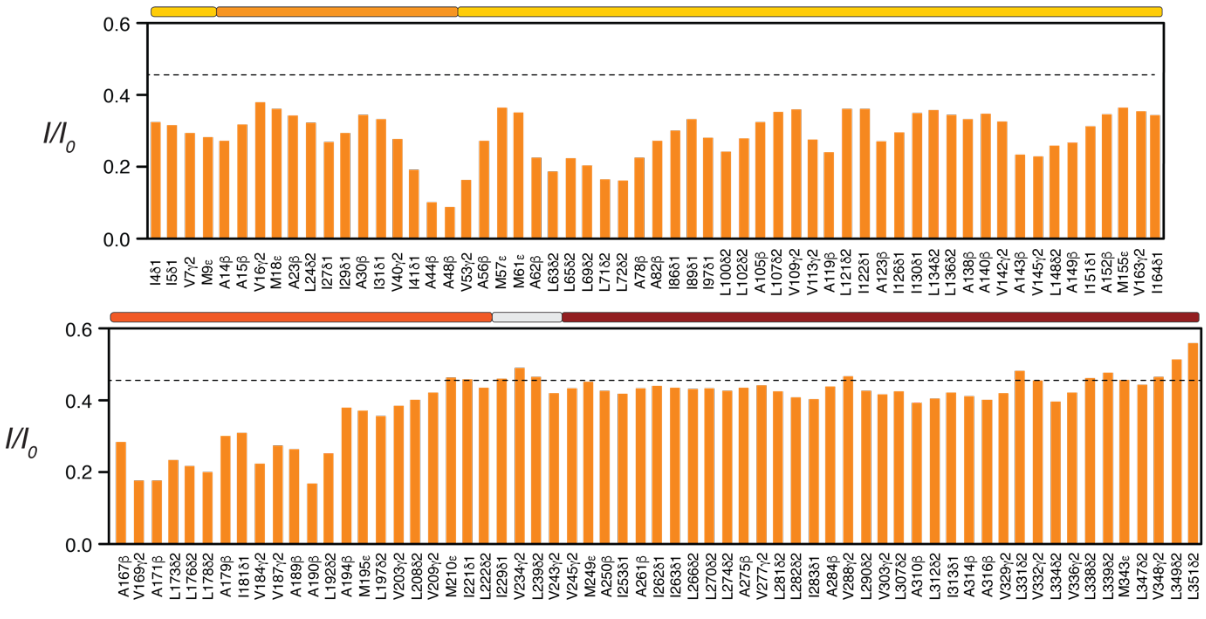

Supplementary Figure S5. The ratio of the individual peak intensities of the indicated methyl groups in the presence of 0.1 equivalents of RNAP. Related to **Figure 7D**.
